# *In vivo* pharmacokinetic evaluation of molnupiravir in patients with severe chronic kidney disease

**DOI:** 10.3389/fphar.2025.1696197

**Published:** 2025-11-18

**Authors:** Yuanyuan Wang, Qiong Chen, Xuan Liu, Ling Zhang, Ming Chen, Shuowen Wang, Man Yang, Guorong Fan, Qiuling Fan

**Affiliations:** 1 Department of Pharmacy, Shanghai General Hospital, Shanghai Jiao Tong University School of Medicine, Shanghai, China; 2 Department of Nephrology, Shanghai General Hospital, Shanghai Jiao Tong University School of Medicine, Shanghai, China

**Keywords:** molnupiravir, pharmacokinetic evaluation, chronic kidney disease, COVID-19, N4-hydroxycytidine

## Abstract

**Introduction:**

Molnupiravir (MPV) is an oral, potent ribonucleoside analog that inhibits the replication of SARS-CoV-2 and is indicated for the treatment of adults with COVID-19. Patients with COVID-19 are at an elevated risk of adverse outcomes, underscoring the need for precise pharmacokinetic (PK) data to guide safe drug use. For MPV and its active metabolite, N4-hydroxycytidine (NHC), renal excretion is not the primary elimination pathway. Consequently, renal impairment has minimal impact on their PK profiles, and no dose adjustment is recommended for patients with mild to moderate renal impairment. However, there are no relevant pharmacokinetic studies in patients with severe renal insufficiency (eGFR<30 mL/min/1.73 m^2^) or patients requiring dialysis. This study aims to explore the plasma concentration of MPV in patients with severe renal insufficiency, providing a basis for rational clinical use of drugs.

**Methods:**

Liquid chromatography-tandem mass spectrometry (LC-MS/MS) was used to detect the plasma concentrations of MPV and NHC. All analytes were extracted by protein precipitation using acetonitrile at a ratio equivalent to 3:1, and QMPV and QNHC were evaluated by calculating the ratio of plasma concentrations.

**Results:**

A total of four patients with stage 4 or 5 chronic kidney disease (CKD) were enrolled in the study. All patients received a standard oral dose of molnupiravir (MPV), and plasma samples were collected 12 h post-administration (C_12h_) for analysis. Plasma concentrations of MPV itself were consistently low at 12 h post-dose. In contrast, the 12-h plasma concentration of N4-hydroxycytidine (NHC, the active metabolite of MPV) in patients with severe renal insufficiency (stage 4/5 CKD) was significantly higher than that in reference populations: compared with healthy subjects (NHC C_12h_: 16.7 ng/mL) and patients with mild to moderate renal impairment (NHC C_12h_: 31.1 ng/mL), the NHC C_12h_ in the severe renal insufficiency group ranged from 43 to 1,600 ng/mL. Notably, the patient with stage 5 CKD exhibited the highest NHC plasma concentration (1,600 ng/mL) at 12 h post-dose, which remained at a persistently elevated level.

**Conclusion:**

MPV was rapidly hydrolyzed to NHC in the body and maintained at a low level. The NHC is significantly higher than that of patients with mild to moderate symptoms, especially those with stage 5 chronic kidney disease. The blood drug concentration is equivalent to Cmax, which suggests that when used clinically in patients with uremia, the dosing interval should be adjusted to avoid drug accumulation and occurrence of AEs.

## 1 Introduction

Molnupiravir (MPV) is an oral antiviral drug originally developed for influenza but later evaluated as a treatment for COVID-19 (Jayk Bernal et al., 2022). MPV is a small-molecule ribonucleoside prodrug of N-hydroxycytidine (NHC), which is taken up by cells and phosphorylated to form the active ribonucleoside triphosphate (NHC-TP). NHC-TP is incorporated into SARS-CoV-2 RNA by the viral RNA polymerase, leading to the accumulation of errors in the viral genome, thereby inhibiting replication (Butler et al., 2023).

MPV is available for oral use only in 200 mg capsules. The existing recommended dose for moderate to severe COVID-19 patients is 800 mg (4 capsules) every 12 h with or without food for 5 days (Saravolatz et al., 2023). Studies have showed that MPV and NHC are not substrates, inhibitors, or inducers of multiple CYP enzymes, human P-glycoprotein (P-gp) or assessed transport proteins. Therefore, no drug interactions with MPV have been found (Saravolatz et al., 2023). What’s more, NHC is cleared via metabolism to cytidine and/or uridine through pyrimidine metabolic pathways, which was not significantly eliminated by renal clearance when evaluated in mild to moderate renal impairment (Syed, 2022). Thus, no dose adjustment is required in patients with mild renal impairment.

Previous studies have confirmed that MPV does not accumulate in CKD patients with COVID-19, and there is no increased risk of adverse events (AEs) in patients with CKD stage 4, 5 and end-stage kidney disease (Chen et al., 2024; Cho et al., 2023; Dufour et al., 2023). However, limited data exist on the concentration of MPV and NHC in patients with eGFR <30 mL/min/1.73 m^2^ and receiving dialysis. Our study focused on those patients with eGFR less than 30 mL/min/1.73 m^2^ or receiving dialysis to explore the impact of renal insufficiency on MPV pharmacokinetics.

## 2 Methods

### 2.1 Subjects

Eligible patients were are those with eGFR less than 30 mL/min/1.73 m^2^ or receiving dialysis and treated with MPV (800 mg). The pharmacokinetic parameters in healthy volunteers and patients without renal impairment were from the drug instructions.

### 2.2 Blood specimen collection and bioanalysis

Blood specimens were collected after 12 h after dosing on Day 5. Stock solutions were prepared from their respective reference standards in 50% acetonitrile to obtain a final concentration of 1 mg/mL and stored at −80 °C until use. Working solutions, containing varying concentrations of MPV and NHC, were prepared by diluting this stock solution in 50% acetonitrile to produce several concentrations which were then spiked into drug-free plasma. Using Ribavirin as internal standard and the internal standard (IS) working solutions for plasma were prepared in 50% acetonitrile.

Plasma working calibration standards of MPV and NHC were prepared from the several concentrations above by diluting in the appropriate volume of blank, drug-free plasma to produce a final concentration of 1.0, 5.0, 10.0, 50.0, 100, 500, 1,000 and 2,500 ng/mL.

Quality control (QC) samples were prepared from the MPV and NHC primary stock. These consisted of high QC (HQC: 2000 ng/mL of MPV and NHC), medium QC (MQC: 200 ng/mL of MPV and NHC), low QC (LQC: 2.0 ng/mL of MPV and NHC) and the lower limit of quantification (LLOQ: 1.0 ng/mL of MPV and NHC) for plasma.

Total MPV and NHC concentrations were determined by using a quality control-validated method based on LC-MS/MS (6460 LC/TQ, Agilent Corp.). Control of hardware and processing of data were performed utilizing Mass Hunter Workstation (Agilent, American). 10 μL IS solution was spiked into 100 μL plasma samples. All the analytes were extracted by protein precipitation using acetonitrile at an equivalent 3:1 ratio as used to extract and stable the clinical samples. MPV diffusion (QMPV) and NHC diffusion (QNHC) were assessed by calculating the ratio of plasma concentrations.

## 3 Results

A total of four patients were included in this study, and the basic characteristics were showed in Table 1. The included patients were diagnosed with chronic kidney disease (CKD) and combined with other diseases. One patient had CKD G3 (eGFR, 32 mL/min/1.73 m^2^), and two had CKD G4 (eGFR, 26 and 22 mL/min/1.73 m^2^) (Table 1). Patients four had CKD G5 (eGFR, 6 mL/min/1.73 m^2^) and was under peritoneal dialysis. Patient 1’s eGFR was close to 30 and showed significant differences before and after medication, therefore was also included in the study. They all received MPV at a dosage of 800 mg twice daily for 5 days (given after hemodialysis on dialysis day) for mild-to-moderate COVID-19.

**TABLE 1:** Baseline and COVID-19 characteristics of patients treated with MPV.

Patients characteristics	Patient 1	Patient 2	Patient 3	Patient 4
Age (years)	74	65	70	85
Sex	Female	Female	Female	Male
Comorbidities	ANCN-associated vasculitis	Chronic glomerulonephritis	Diabetic nephropathy	Hypertension
	Hypertension	Hypertension	Renal anemia	Renal anemia
	Gallbladder stones	Gout	Diabetic foot	Hyperuricemia
	Cholecystitis	Liver insufficiency	Diabetic retinopathy	Benign prostatic hyperplasia
	Arrhythmia	Left kidney atrophy		
Medication use	Ferrous succinate sustained release tablets	Ferrous succinate sustained release tablets	Nifedipine	Amlodipine besylate
	Roxadustat	Febuxostat	Sacubitril valsartan sdium tablets	Roxadustat
	Recombinant human erythropoietin injection (CHO cell)	Prednisone acetate tablets	Insulin	Ferrous succinate sustained release tablets
	Pravastatin sodium tablets	Estazolam		PPI
	Methylprednisolone			
	Rituximab injection			
	Ampicillin sodium and sulbactamsodium			
CKD stage	Stage 3b	Stage 4	Stage 4	Stage 5
eGFR (mL/min/1.73 m^2^)	35.63	27.63	23.88	6.97
Peritoneal dialysis	No	No	No	Yes
Baseline labs				
Blood pressure (mmHg)	117/99	191/95	183/110	130/78
Serum albumin (g/L)	29.5	36.7	25	27
Hemoglobin (g/L)	78	116	98	111
Platelet (109/L)	384	213	144	129
Cr (μmol/L)	134.8	174.6	198.1	631.6
AST (U/L)	10.74	15.48	21.5	12.75
ALT (U/L)	4.3	12.3	14.6	7.5
Uric acid (μmol/L)		490.8	334.5	461.8

The results showed that the concentration of MPV was very low in the blood samples of patients, which rapidly degraded into NHC after oral administration. Notably, patients with low eGFR (<30 mL/min/1.73 m^2^) value exhibited higher NHC concentration than subjects with normal kidney function (C_12h_: 31.1 ng/mL) and healthy volunteers (C_12h_: 16.7 ng/mL). Especially, patients 4 (CKD G5, eGFR, 6.97 mL/min/1.73 m^2^), who was under peritoneal dialysis, had the highest NHC concentration (1600.4 ng/mL) (Table 2).

**TABLE 2:** Concentration of MPV/NHC AND adverse events of patients treated with MPV.

Patients characteristics	Patient 1	Patient 2	Patient 3	Patient 4
MPV (ng/mL)	2.2	1.91	2.23	5.04
NHC (ng/mL)	68.4935	148.9662	75.9679	1600.3807
Adverse events reported				
Gastrointestinal upset	No	Yes	Yes	No
Dry mouth	No	No	No	No
Dysgeusia	No	No	No	No
Fatigue	No	No	Yes	No
Dizziness	No	No	No	Yes
Dyspnea	No	No	No	No
Myalgia	No	No	No	No
Insomnia	No	No	Yes	No
Delirium	No	No	No	No
Gout	No	No	No	No
Worsening kidney function	No	No	Yes	No
Unresolved COVID-19 symptoms	No	No	Yes	Yes
AE leading to treatment discontinuation	No	No	No	No

## 4 Discussion

To our knowledge, the higher the blood concentration of a drug, the greater the intensity of the drug’s effect. When it is higher than the minimum concentration that produces side effects, toxic side effects may occur. If it is lower than the minimum concentration that produces a therapeutic effect, concentrations may be ineffective or may lead to resistance. In this study, patient two developed sudden hepatic insufficiency and had gastrointestinal upset, which is the common adverse event (AE to MPV, including nausea and vomiting. MPV does not affect liver function, and patient two also took febuxostat, which affects liver functions. After discontinuing febuxostat and using hepatoprotective drugs, the patient’s liver function gradually recovered. Notably, patient three had gastrointestinal upset, fatigue, worsening kidney function, and unresolved COVID-19 symptoms. Due to the ineffectiveness of MPV, it was switched to Paxlovid (Nirmatrelvir/Ritonavir). Although the concentration of NHC was 50 times higher than that of subjects with normal kidney function, patient four still had COVID-19 infection and then took Paxlovid after the ineffectiveness of MPV. And patient four also reported AE of dizziness.

While the primary elimination route of NHC may be hepatic metabolism, significant accumulation of NHC is still observed in patients with advanced CKD. Beyond renal excretion, several key factors may contribute to the altered pharmacokinetics (PK) of NHC in this population, as elaborated below: First, the uremic state in advanced CKD can profoundly impair the function of drug-metabolizing enzymes and transporters. Uremic toxins can directly inhibit the activity of cytochrome P450 enzymes and uridine diphosphate-glucuronosyltransferases (UGTs)—families of enzymes likely involved in the metabolic clearance of NHC. Thus, the efficiency of its non-renal elimination (e.g., hepatic and intestinal clearance) can be substantially reduced. Second, advanced CKD is closely associated with marked gut microbiota dysbiosis and impaired intestinal barrier integrity. These pathological changes can perturb the enterohepatic circulation of drugs and their metabolites. A plausible mechanism is that NHC or its conjugated metabolites are excreted into the bile, then deconjugated by dysregulated gut flora in the intestinal lumen, and subsequently reabsorbed into the systemic circulation. This cycle of biliary excretion, deconjugation, and reabsorption leads to prolonged systemic exposure to NHC and eventual drug accumulation. The metabolic disturbances in advanced CKD are widespread and affect core biochemical pathways. Uremia leads to the retention of a wide array of nitrogenous waste products. Some of these may be structural analogs of endogenous pyrimidines (e.g., orotic acid, uracil).

In this study, there seems a connection between high MHC concentration and AEs in MPV-treated CKD and dialysis patients. However, the level of blood drug concentration is not directly proportional to the occurrence of AEs. Because patients also took other drugs at the same time, we cannot rule out AEs caused by other medications. Even high blood drug concentration cannot relieve COVID-19 symptoms. But we still detect excessively high blood drug concentration and pay close attention to AEs to promptly adjust medication intervals and doses.

## Data Availability

The original contributions presented in the study are included in the article/supplementary material, further inquiries can be directed to the corresponding authors.

## References

[B1] Butler C. C. Hobbs F. D. R. Gbinigie O. A. Rahman N. M. Hayward G. Richards D. B. (2023). Molnupiravir plus usual care versus usual care alone as early treatment for adults with COVID-19 at increased risk of adverse outcomes (PANORAMIC): an open-label, platform-adaptive randomised controlled trial. Lancet 401 (10373), 281–293. 10.1016/S0140-6736(22)02597-1 36566761 PMC9779781

[B2] Chen C. C. Huang C. Y. Wu J. Y. Liu M. Y. Chuang M. H. Liu T. H. (2024). Clinical effectiveness of oral antiviral agents for treating non-hospitalized COVID-19 patients with chronic kidney disease. Expert Rev. Anti Infect. Ther. 22 (8), 705–712. 10.1080/14787210.2024.2334052 38525673

[B3] Cho W. J. Harden D. Moreno D. Dinulos J. E. Hanna P. E. Wang Q. (2023). Oral antiviral therapies for COVID-19 in patients with advanced chronic kidney disease or kidney failure. Nephrol. Dial. Transpl. 38 (8), 1912–1914. 10.1093/ndt/gfad058 36948600 PMC10387392

[B4] Dufour I. Devresse A. Scohy A. Briquet C. Georgery H. Delaey P. (2023). Safety and efficiency of molnupiravir for COVID-19 patients with advanced chronic kidney disease. Kidney Res. Clin. Pract. 42 (2), 275–278. 10.23876/j.krcp.22.194 37037486 PMC10085722

[B5] Jayk Bernal A. Gomes D. S. M. M. Musungaie D. B. Kovalchuk E. Gonzalez A. Delos Reyes V. (2022). Molnupiravir for oral treatment of Covid-19 in nonhospitalized patients. N. Engl. J. Med. 386 (6), 509–520. 10.1056/NEJMoa2116044 34914868 PMC8693688

[B6] Saravolatz L. D. Depcinski S. Sharma M. (2023). Molnupiravir and nirmatrelvir-ritonavir: oral coronavirus disease 2019 antiviral drugs. Clin. Infect. Dis. 76 (1), 165–171. 10.1093/cid/ciac180 35245942 PMC9383515

[B7] Syed Y. Y. (2022). Molnupiravir: first approval. Drugs 82 (4), 455–460. 10.1007/s40265-022-01684-5 35184266 PMC8858220

